# A Path-Planning Method Based on Improved Soft Actor-Critic Algorithm for Mobile Robots

**DOI:** 10.3390/biomimetics8060481

**Published:** 2023-10-10

**Authors:** Tinglong Zhao, Ming Wang, Qianchuan Zhao, Xuehan Zheng, He Gao

**Affiliations:** 1 School of Information and Electrical Engineering, Shandong Jianzhu University, Jinan 250101, China; zhaotinglong9809@163.com (T.Z.); zxh656@126.com (X.Z.); gaohe118@163.com (H.G.); 2 Department of Automation, Tsinghua University, Beijing 100018, China; zhaoqc@tsinghua.edu.cn; 3 Shandong Zhengchen Technology Co., Ltd., Jinan 250000, China

**Keywords:** mobile robot, path planning, reinforcement learning, soft actor-critic, hindsight experience replay

## Abstract

The path planning problem has gained more attention due to the gradual popularization of mobile robots. The utilization of reinforcement learning techniques facilitates the ability of mobile robots to successfully navigate through an environment containing obstacles and effectively plan their path. This is achieved by the robots’ interaction with the environment, even in situations when the environment is unfamiliar. Consequently, we provide a refined deep reinforcement learning algorithm that builds upon the soft actor-critic (SAC) algorithm, incorporating the concept of maximum entropy for the purpose of path planning. The objective of this strategy is to mitigate the constraints inherent in conventional reinforcement learning, enhance the efficacy of the learning process, and accommodate intricate situations. In the context of reinforcement learning, two significant issues arise: inadequate incentives and inefficient sample use during the training phase. To address these challenges, the hindsight experience replay (HER) mechanism has been presented as a potential solution. The HER mechanism aims to enhance algorithm performance by effectively reusing past experiences. Through the utilization of simulation studies, it can be demonstrated that the enhanced algorithm exhibits superior performance in comparison with the pre-existing method.

## 1. Introduction

The proliferation of mobile robots across several industries is a direct consequence of advancements in science and technology. The significance of path planning in enabling mobile robots to achieve autonomous navigation has been widely recognized and has experienced substantial advancements and increased attention in recent times [1]. The primary objective of path planning is to identify a path that is both safe and free from collisions, while also minimizing the distance traveled, within a complex environment. This enables a robot to successfully navigate from its present state to a desired target state [2].

The issue of path planning can be categorized into two basic components: global path planning and local path planning. These components rely on the robot’s acquisition of information pertaining to its immediate surroundings. Global route planning refers to the entirety of the environment information that is known to the robot, whereas local path planning pertains to the knowledge about the environment that is only partially known or completely unknown to the robot [3]. The primary algorithms for global path planning can be categorized into two groups: traditional algorithms, such as the A* algorithm [4], the Dijkstra algorithm [5], rapidly exploring random tree (RRT) [6], and probabilistic road map (PRM) [7], and intelligent algorithms, including the ant colony optimization (ACO) algorithm [8] and genetic algorithm (GA) [9], and the particle swarm optimization (PSO) algorithm [10]. The primary algorithms utilized for local path planning can be classified into two categories: traditional algorithms, such as artificial potential field (APF) [11], the dynamic window approach (DWA) [12], and the time elastic band (TEB) algorithm [13], and artificial intelligence algorithms, including the neural network (NN) algorithm [14] and the reinforcement learning (RL) algorithm, among others. Global path-planning algorithms are particularly suitable for performing path searches in static environments, where it is essential to have comprehensive knowledge of the environment’s map information. Nevertheless, in intricate settings, these algorithms often exhibit extended and inefficient planning durations, and they are susceptible to becoming trapped in local optima. In real-world situations, it is frequently observed that environmental data may exhibit a lack of comprehensiveness. Simultaneously, the computational complexity experiences a significant augmentation as a result of the necessity to explore the entirety of the map, rendering it incapable of fulfilling real-time demands. As a result, the need for real-time path planning arises. Nevertheless, conventional local path-planning techniques demonstrate shortcomings in terms of their capacity to adapt to the environment, efficiency, and vulnerability to local optima. Consequently, they are unable to produce the path that is globally optimal. Hence, the significance of a path-planning approach that incorporates autonomous learning and decision-making capabilities cannot be overstated.

Reinforcement learning (RL) is an algorithm characterized by autonomous learning, which has gained significant prominence in recent years. In contrast with conventional algorithms that typically rely on the availability of complete or partial environmental information, reinforcement-learning-based algorithms exhibit the unique characteristic of not requiring prior knowledge of the environmental information. In order to adapt to unfamiliar surroundings, individuals have the ability to modify their activities by continuously receiving feedback from interactions with the environment. From autonomous learning, they are able to make informed judgments that lead to greater rewards, ultimately enabling them to strategize and determine the most ideal path [15]. Duguleana et al. employed a hybrid approach that integrated Q-learning and neural networks to accomplish path planning for mobile robots [16]. The proposed approach successfully accomplishes autonomous obstacle avoidance for both stationary and moving objects, regardless of whether global information is available or not. Maoudj et al., introduced a novel approach to path optimization using Q-learning. Their method involves equipping the robot with a priori knowledge through the utilization of a new reward function. This approach facilitates accelerated learning and enhances the optimization process, leading to fast convergence [17]. In their study, Pei et al. integrated a heuristic search strategy and simulated an annealing mechanism into an enhanced version of the Dyna-Q algorithm. This integration resulted in enhancements to both the global search rate and the learning efficiency of path planning, as reported in their research [18]. Wen et al. introduced a hierarchical SARSA approach that utilizes topological maps [19]. This method involves generating the topological area using a dynamic growth algorithm. Additionally, the authors incorporate the artificial potential field method and topological maps to initialize the Q-tables separately. This integration results in improved convergence of the algorithm.

Deep reinforcement learning (DRL) is a more effective way of learning by combining the neural network of deep learning with the reinforcement learning algorithm and using the neural network as a function approximator to represent the value function or the policy function so that the deep reinforcement learning (DRL) so constituted is a more effective way of learning. Yang et al. solved the problem of a slow rate of convergence and high randomness in path planning by adding prior knowledge and rules to the deep-Q network (DQN) algorithm and improved the efficiency of path planning [20]. Yang et al. proposed a global path-planning algorithm based on dual-deep-Q networks (DDQNs), which combines the a priori knowledge and action masking methods, and adapts the optimized paths by adjusting the reward functions for different tasks [21]. Sasaki et al. proposed an asynchronous-advantage-based asynchronous advantage actor-critic (A3C) algorithm, which is able to adapt to dynamic environments and thus perform path planning [22]. Chen et al. combined the preferential experience replay (PER) technique with the soft actor-critic (SAC) algorithm for deep reinforcement learning for path planning, which improves the sample utilization rate and the success rate of path planning [23]. Xu et al. decoupled the decoupled hybrid action space, reduced robot crosstalk using a centralized training decentralized execution framework, and optimized the soft actor-critic (SAC) algorithm to improve the convergence and robustness of the algorithm [24]. Tian et al. added hierarchical learning (HL) and particle swarm optimization (PSO) to an improved deep policy gradient (DDPG) algorithm to improve path planning by setting the buffer to improve the path accuracy, which, in turn, improves the convergence speed and accuracy of path planning [25]. Gao et al. combined the traditional algorithm probabilistic roadmap (PRM) with twin-delayed depth deterministic policy gradient (TD3), which improves the adaptability of the model and reduces the training time through dimensionality reduction to improve the efficiency of path planning [26]. Cheng et al. used the deep policy gradient algorithm (DDPG) to establish control laws for linear and steering speeds and implemented a tracking obstacle avoidance control method by redesigning the state and reward functions [27]. Despite the advancements made in reinforcement learning, the aforementioned methods still possess some limitations. These include unstable training, inadequate convergence, incentive overestimation, limited adaptability to environmental conditions, and suboptimal utilization of samples in real-world scenarios.

In this paper, we propose an improved deep reinforcement learning SAC method to apply it to path planning for mobile robots. The main contributions of this paper are summarized as follows:

- In this study, we provide a novel deep reinforcement learning method based on the soft actor–critic (SAC) framework for the purpose of path planning in settings with unknown characteristics. The algorithm has been modified to accommodate a continuous action space and operates as an offline method. The introduction of maximum entropy is employed to mitigate the issue of local optimality, hence enhancing the system’s resistance to interference.
- Furthermore, the hindsight experience replay (HER) algorithm is proposed as a solution to address the challenges of reward scarcity and the sluggish training pace seen in goal-oriented reinforcement learning algorithms. HER achieves this by recalculating reward values and efficiently using the knowledge gained from unsuccessful experiences during the training process.
- Third, the simulation experiments of path planning with environmental maps verify that the new algorithm HER-SAC can effectively perform path planning and improve the training speed and convergence of the algorithm.

The subsequent sections of this paper are structured in the following manner. Section 2 provides an overview of the theoretical underpinnings of the path-planning method, encompassing two key components: reinforcement learning and the soft actor–critic (SAC) algorithm. Section 3 provides an overview of the theoretical foundation and framework of the enhanced algorithm, along with a detailed explanation of the design considerations for the action space, state space, and reward function. Section 4 of this paper provides a comparative analysis of various algorithms for path planning in a simulated environment. The discussion and analysis of the simulation results for path planning are presented in Section 5. Section 6 provides the concluding remarks for this research article.

## 2. Path-Planning Algorithm

The problem of path planning for mobile robots is framed within the context of reinforcement learning. Additionally, an advanced deep reinforcement learning technique called soft actor–critic (SAC) is introduced. This section will be utilized in the subsequent phase to enhance the algorithm.

### 2.1. Enhanced Learning

Reinforcement learning, situated between the supervised and unsupervised learning paradigms, enables the acquisition of knowledge through iterative interactions with the environment, devoid of external supervision or direction. The field of reinforcement learning incorporates concepts and principles derived from the field of bionics in order to develop learning algorithms that are both more efficient and cleverer. One application of reinforcement learning is training intelligent systems to acquire optimal behavioral strategies by emulating the reward mechanisms observed in biological systems. Furthermore, reinforcement learning is influenced by the field of bionics in order to develop learning algorithms that exhibit enhanced adaptability in intricate environments and dynamic circumstances. Hence, the utilization of reinforcement learning for addressing path-planning challenges in unfamiliar contexts seems to be a highly efficient approach.

**Figure 1:**
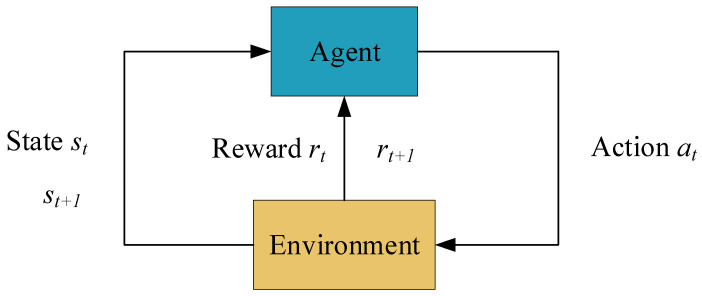
Basic model of reinforcement learning.

Reinforcement learning is an algorithm described using the Markov decision process (MDP), so the path planning of a mobile robot can be reduced to a Markov decision process. The process can be defined as a five-tuple $\left(S,A,P,R,\gamma \right)$, where S denotes the state space, A denotes the action space, P denotes the state transfer probability, R denotes the reward function, and γ denotes the discount factor. Figure 1 shows the process of reinforcement learning.

The robot interacts with the environment as it completes its tasks, using a policy to input actions $a_{t}$ into the environment. The environment receives the action and changes its state from $s_{t}$ to $s_{t+1}$ and returns the reward to $r_{t+1}$ the robot. The robot then chooses the next action based on the reward, and the cycle continues to improve the policy. Eventually, the optimal strategy with the largest cumulative reward is obtained through an iterative loop process. The cumulative reward $G_{t}$ for the whole learning process is defined as:

(1) $$G_{t}=r_{t+1}+\gamma r_{t+2}+\gamma^{2}r_{t+3}+\ldots =\sum_{k=0}^{\infty }\gamma^{k}r_{t+k+1}$$

where $\gamma \in \left[0,1\right]$ is the variable that determines how future rewards are valued. The closer to 1 the γ is means that future rewards are more important.

The strategy of a robot is usually denoted by π. In order to evaluate the value of states and actions, the state value function $V^{\pi }(s_{t})$ and the state–action value function $Q^{\pi }(s_{t},a_{t})$ are introduced as the expected payoffs of the strategy, defined as:

(2) $$V^{\pi }(s_{t})=E_{\pi }[G_{t}|s_{t}]$$

(3) $$Q^{\pi }(s_{t},a_{t})=E_{\pi }[G_{t}|s_{t},a_{t}]$$

The Bellman expectation equation (BEE) for two value functions is defined as:

(4) $$V^{\pi }(s_{t})=E_{\pi }[r_{t}{}_{+1}+\gamma V^{\pi }(s_{t+1})|s_{t}]$$

(5) $$Q^{\pi }(s_{t},a_{t})=E_{\pi }[r_{t+1}+\gamma Q(s_{t+1},a_{t+1})|s_{t},a_{t}]$$

The purpose of reinforcement learning is to find the optimal strategy, and the corresponding value function can be used to compare the advantages and disadvantages of the strategy, so the optimal state value function and the optimal state–action value function can be expressed as:

(6) $$V^{*}(s)=max_{\pi }V^{\pi }(s_{t})$$

(7) $$Q^{*}(s_{t},a_{t})=max_{\pi }Q^{\pi }(s_{t},a_{t})$$

The optimal policy can be obtained by maximizing the optimal value function, so the value function and Bellman’s expectation equation are important components of reinforcement learning on which subsequent reinforcement learning algorithms are based.

### 2.2. Soft Actor-Critic (SAC) Algorithm

The SAC algorithm is a reinforcement learning method that incorporates deep learning techniques, specifically deep learning (DL), and integrates the maximum entropy framework with the actor-critic (AC) framework through the utilization of offline and stochastic strategies. The SAC algorithm possesses a significant advantage over other reinforcement learning algorithms and deep reinforcement learning algorithms due to its notable exploratory capabilities and its ability to adapt effectively to more intricate challenges.

#### 2.2.1. Maximizing Entropy

Entropy is used to indicate the degree of randomness of a random variable, and in the calculation, the variable x obeys the probability distribution P, so the entropy value $H\left(P\right)$ is defined as:

(8) $$H(P)=\underset{x\sim P}{E}[-\log P(x)]$$

The goal of the SAC algorithm with the introduction of maximizing entropy is to not only maximize the cumulative reward but also to make the strategy more random, i.e., maximize the entropy value of the strategy, with the objective function defined as:

(9) $$J(\pi )=\sum_{t=0}^{T}E_{\left(s_{t},a_{t}\right)∼\rho_{\pi }}\left[r\left(s_{t},a_{t}\right)+\alpha H\left(\pi \left(\cdot |s_{t}\right)\right)\right]$$

where π denotes the current policy of the network, $\rho_{\pi }$ denotes the distribution of actions and states under the policy π, T denotes the total number of time steps for the robot to interact with the environment, E denotes the reward expectation of the current state, r denotes the reward value of the current state, $H\left(\cdot \right)$ denotes the entropy value, and α denotes the temperature coefficient, which determines the relative importance of entropy to the reward.

#### 2.2.2. Soft Strategy Iteration

The soft strategy iteration is performed alternately between strategy evaluation and strategy improvement. In strategy evaluation, the value of the strategy is calculated based on maximizing the entropy, and for a fixed strategy, the modified Bellman backup operator for the soft *Q*-value is:

(10) $$\tau_{\pi }Q\left(s_{t},a_{t}\right)=r\left(s_{t},a_{t}\right)+\gamma E_{s_{t+1}}{}_{∼p}\left[V\left(s_{t+1}\right)\right]$$

where the state value function is defined as:

(11) $$V\left(s_{t}\right)=E_{a_{t}}{}_{∼\pi }\left[Q\left(s_{t},a_{t}\right)-log\pi \left(a_{t}|s_{t}\right)\right]$$

Ultimately, the soft policy evaluation converges to the soft *Q*-value of the policy π through $Q^{k+1}=\tau_{\pi }Q^{k}$ iterations.

In strategy improvement, the strategy is improved according to the soft strategy enhancement formula:

(12) $$\pi_{new}=arg\;\underset{\pi^{\prime }\in \Pi }{min}D_{KL}(\pi^{\prime }\left(\cdot |s_{t}\right)||\frac{exp\;\left(Q^{\pi_{old}}\left(s_{t},\cdot \right)\right)}{Z^{\pi_{old}}\left(s_{t}\right)})$$

where KL denotes the Kullback–Leibler scattering, $Z^{\pi_{old}}\left(s_{t}\right)$ normalizes the distribution of *Q*-values, and the strategies, $\pi^{\prime }\in \Pi$, are constrained to be in the parameter space Π, which ensures that all new strategies are stronger than the old ones.

#### 2.2.3. Soft Actor-Critic

The above soft policy iterative process is for the case of tabular setups, for environments with limited state and action spaces. For continuous space, function approximation needs to be introduced. The SAC network consists of five neural networks: the policy network $\pi_{\varphi }(a_{t}|s_{t})$, the state value network $V_{\psi }\left(s_{t}\right)$, the target state value network $V_{\bar{\psi }}\left(s_{t}\right)$, and two soft *Q*-value networks $Q_{\theta }\left(s_{t},a_{t}\right)$, with corresponding parameters φ,ψ,θ. The use of the double-*Q* structure alleviates the case of overestimation of the *Q*-value and applies stochastic gradient descent to the objective function. Therefore, the objective function of the soft state value function is defined as:

(13) $$J_{V}(\psi )=E_{s_{t}∼D}\left[\frac{1}{2}{\left(V_{\psi }\left(s_{t}\right)-E_{a_{t}∼\pi_{\phi }}\left[Q_{\theta }\left(s_{t},a_{t}\right)-\log\pi_{\phi }\left(a_{t}|s_{t}\right)\right]\right)}^{2}\right]$$

The gradient is:

(14) $$\hat{\nabla }\psi J_{V}\left(\psi \right)=\nabla_{\psi }V_{\psi }\left(s_{t}\right)\left(V_{\psi }\left(s_{t}\right)-Q_{\theta }\left(s_{t},a_{t}\right)+log\pi_{\varphi }\left(a_{t}|s_{t}\right)\right)$$

The objective function of the soft *Q*-value function is defined as:

(15) $$J_{Q}\left(\theta \right)=E_{\left(s_{t},a_{t}\right)∼D}\left[\frac{1}{2}\right.Q_{\theta }\left(s_{t},a_{t}\right)-\left.\hat{Q}{\left(s_{t},a_{t}\right)}^{2}\right]$$

Of these,

(16) $$\hat{Q}\left(s_{t},a_{t}\right)=r\left(s_{t},a_{t}\right)+\gamma E_{s_{t+1}∼p}\left[V_{\bar{\psi }}\left(s_{t+1}\right)\right]$$

The gradient is:

(17) $${\hat{\nabla }}_{\theta }J_{Q}(\theta )=\nabla_{\theta }Q_{\theta }\left(a_{t},s_{t}\right)\left(Q_{\theta }\left(s_{t},a_{t}\right)\right.-r\left(s_{t},a_{t}\right)-\left.\gamma V_{\bar{\psi }}\left(s_{t+1}\right)\right)$$

The objective function of the strategy update is:

(18) $$J_{\pi }\left(\varphi \right)=E_{s_{t}∼D}\left[D_{KL}\left(\pi_{\varphi }\left(\cdot |s_{t}\right)||\frac{exp\left(Q_{\theta }\left(s_{t},\cdot \right)\right)}{Z_{\theta }\left(s_{t}\right)}\right)\right]$$

Since the SAC algorithm outputs Gaussian-distributed means and standard deviations and the process of sampling the action is not derivable, the actions need to be sampled using a reparameterization technique, denoted as:

(19) $$a_{t}=f_{\varphi }\left(\epsilon_{t};s_{t}\right)$$

where $\epsilon_{t}$ is the input noise vector, and bringing the above Equation (19) into the objective function (18), the rewritten objective function is:

(20) $$J_{\pi }\left(\varphi \right)=E_{s_{t}∼D}{{}_{,}}_{\epsilon_{t}∼N}\left[log\pi_{\varphi }\left(f_{\varphi }\left(\epsilon_{t};s_{t}\right)|s_{t}\right)-Q_{\theta }\left(s_{t},f_{\varphi }\left(\epsilon_{t};s_{t}\right)\right)\right]$$

## 3. Improvement of SAC Algorithm

Since the SAC algorithm requires the robot to obtain the final reward after reaching the goal, the reward is sparse, the sample sampling is less efficient, and the training time is longer. In order to improve the efficiency of sample use, this paper designed the HER-SAC algorithm, which introduces hindsight experience replay (HER) [28] into the SAC algorithm. The hindsight experience replay algorithm incorporates the concept of bionics to enhance the learning efficiency and decision-making capabilities of intelligent entities. This is achieved by emulating the learning and memory mechanisms observed in biological systems. The storage and playback mechanism employed in the hindsight experience replay algorithm can be likened to the memory and recall process observed in biological systems. Through the continuous playback of experiences, an intelligent entity can acquire additional knowledge and experience, thereby enhancing its adaptability and optimization capabilities. In the initial algorithm, if the robot does not reach the goal in a sequence, the path sequence is a failure, and since the robot does not complete the task, this means that the robot is unable to learn experience from this sequence. To solve this problem of wasted experience, HER selects a new goal $g^{\prime }$ reusing the whole trajectory.

The core idea of HER is that in the off-policy algorithm, the initial samples are first collected through the interaction between the robot and the environment. A trajectory sample obtained from an episode interaction $s_{1},s_{2},\cdots ,s_{T}$ is stored in the experience pool as a transition. The transition is $\left(s_{t},a_{t},r_{t},s_{t+1}\right)$, which is based on the original target g, and can also be written as $\left(s_{t}∥g,a_{t},r_{t},s_{t+1}∥g\right)$. Then, a set of additional targets *G* is sampled, from which $g^{\prime }\left(g^{\prime }\in G\right)$ is selected, at which point the transition is changed from the original to $\left(s_{t}∥g^{\prime },a_{t},r_{t}^{\prime },s_{t+1}∥g^{\prime }\right)$ based on $g^{\prime }$, and the new transition is stored in the experience pool, and the strategy is trained using the newly obtained tuple. When obtaining a new goal, the future method is used to select it, choosing some state that is in the same trajectory as the rewritten tuple and after it in time as the new goal.

**Figure 2:**
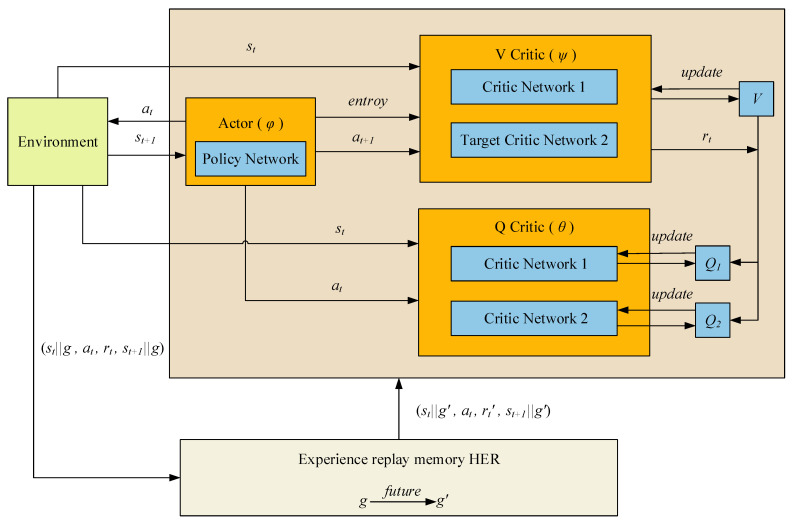
Network structure of the HER-SAC algorithm.

By replacing the experience pool of the original SAC with the experience pool constructed with HER, the new HER-SAC algorithm can utilize the experience of failures, thus making the algorithm more learnable as a whole, and the network structure of the algorithm is shown in Figure 2.

The network structure of the HER-SAC algorithm consists of Actor network, two Q Critic networks and two V Critic networks. Among them, the Actor network interacts with the environment and outputs the probability distribution of actions; the Q Critic network is used to output the state action value function (*Q*-value); and the V Critic network is used to output the state value function (*V*-value).The relationship between the Q network and the V network can be described by the Bellman equation, which allows for simultaneous updating of the networks and learning of the estimates of the *Q*-value and the *V*-value by sharing parameters. The HER-SAC algorithm also includes an important component, the HER module, which is used to extract experience from the experience playback buffer and generate new experience by modifying the target state to take the old experience acquired from the environment and then feeding it back to update the network.

Through a comparative analysis between the enhanced algorithm and the pre-existing algorithm, an examination of their respective merits and drawbacks, as well as an assessment of their complexities, revealed that the enhanced algorithm exhibits adaptability to higher dimensions, the ability to dynamically regulate parameters, enhanced robustness, and improved convergence and stability through the resolution of sparse rewards. Simultaneously, it effectively mitigates the issues arising from parameter sensitivity while maintaining a modest level of complexity, as seen in Table 1.

The motion of the robot generates actions and states, and by analyzing the robot, its actions are decomposed into up and down and left and right. At each moment, the robot can choose to move $\left[-1,\;1\right]$ in the vertical and horizontal directions, respectively, as an action at that moment. As a result, the next state of the robot produces changes in the four directions of up, down, left, and right relative to the previous state. The sizes of the action space and state space are set as in Table 2.

Every movement and state of the robot generates rewards, and the reward function is what determines how fast and how well the algorithm converges, and a proper reward function helps to improve the performance of the algorithm. The rewards generated throughout the robot’s movement are categorized into the following parts: (1) If an obstacle or boundary is encountered, a large negative reward is given ($r_{1}$). (2) A positive reward is given if the goal is reached ($r_{2}$). (3) A smaller negative reward is given if none of the above occurs ($r_{3}$).

(21) $$\begin{array}{l} Reward\left\{\begin{array}{l} r_{1},\;obstacles\\ r_{2},\;goal\\ r_{3},\;other \end{array}\right. \end{array}$$

## 4. Path-Planning Simulation Experiment

### 4.1. Simulation Experiment Environment

To validate the feasibility of the algorithms, the environment maps and algorithms were written in Python. The experimental equipment was a desktop computer with an Intel(R) Core(TM) i7-6700 CPU @ 3.40 GHz 3.41 GHz and 8.00 GB of RAM. In this study, it was postulated that the experimental setting consisted of a 20 × 20 two-dimensional planar space. Within this space, various static obstacles of varying sizes were distributed, each occupying a fixed position and being immovable. The mobile robot under consideration was assumed to be a two-dimensional mass point, with no consideration given to its physical appearance or shape. Additionally, in order to simulate real-world conditions, both the robot and the obstacles maintained a certain distance from each other during the process of path planning.

**Figure 3:**
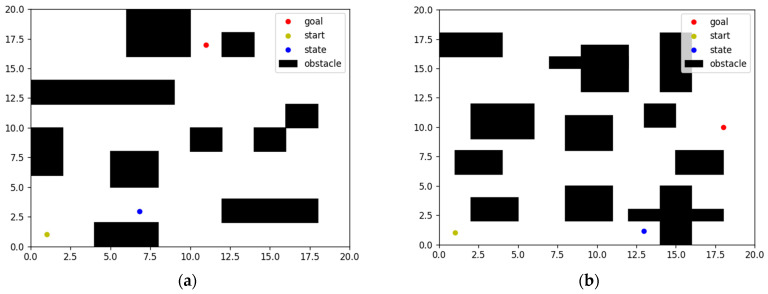
Map of different environments. (**a**) Map1. (**b**) Map2.

As shown in Figure 3, there are two maps with different environments, where the black rectangle is the obstacle, the yellow dot is the start point, the red dot is the target point, the blue dot is the current state, and the blank area is the actionable area.

The parameters of the improved HER-SAC algorithm are shown in Table 3.

### 4.2. Comparison of Algorithms

In order to verify the feasibility of the algorithms, three algorithms, DDPG, SAC, and HER-SAC, were used for path planning and a comparative analysis of the same experimental environment.

**Figure 4:**
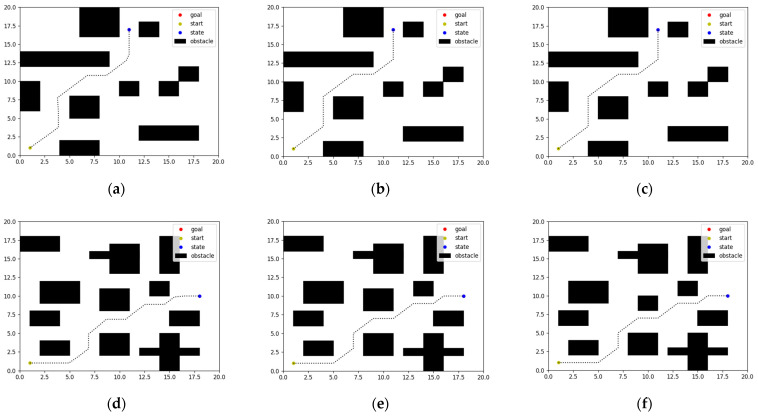
Path planning with different algorithms in different environments. (**a**) DDPG. (**b**) SAC. (**c**) HER-SAC. (**d**) DDPG. (**e**) SAC. (**f**) HER-SAC.

The paths planned by the three algorithms after learning and training iterations are shown in Figure 4, where the black dotted line is the path, the dot (1, 1) is the starting point, and the dots (11, 17) and (18, 10) are the goal points. It can be seen that the three algorithms finally make the state reach the goal point by avoiding obstacles from the starting point.

**Figure 5:**
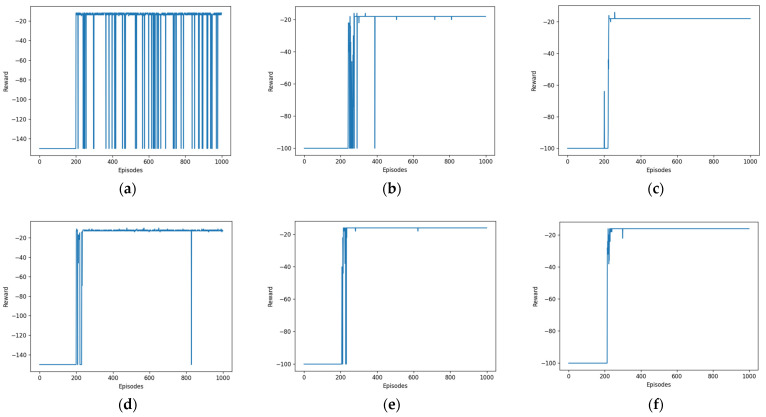
Reward per round curves for different algorithms. (**a**) DDPG. (**b**) SAC. (**c**) HER-SAC. (**d**) DDPG. (**e**) SAC. (**f**) HER-SAC.

As depicted in Figure 5, the graph displays the reward associated with each of the 1000 rounds, represented by the blue curve. The reward mechanism is designed in such a way that it yields a negative reward. The initial stages of the reward curve exhibit minimal variation as a result of encountering the maximum sequence length, failure to achieve the objective, and encountering obstacles. Following multiple iterations of exploration, the reward curve undergoes modifications as a consequence of distinct benefits associated with approaching an obstacle versus reaching a goal. After undergoing numerous additional iterations of learning, the reward curve gradually approaches a state of convergence and ultimately tends to converge.

Upon doing a comparative analysis of various settings, it becomes evident that the convergence of the deep deterministic policy gradient (DDPG) exhibits notable improvement, although it remains incompletely converged. In contrast, both soft actor-critic (SAC) and hindsight experience replay–soft actor-critic (HER-SAC) demonstrate considerably stronger convergence in comparison with DDPG.

**Figure 6:**
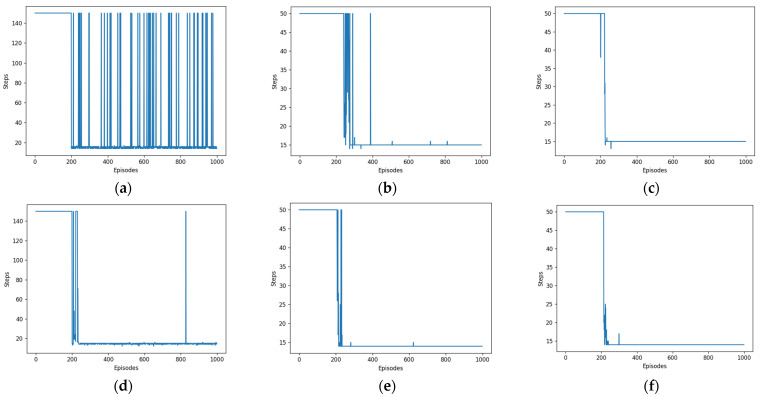
Steps per turn curves for different algorithms. (**a**) DDPG. (**b**) SAC. (**c**) HER-SAC. (**d**) DDPG. (**e**) SAC. (**f**) HER-SAC.

As shown in Figure 6, the blue arcs depicted in the graph represent the number of steps associated with each of the 1000 rounds. The presentation can be considered as the antithesis of the reward curve, with both ultimately converging.

The comparison of results obtained from the execution of three algorithms in varying environments is presented in Table 4.

## 5. Discussion

Our work focused on the examination of algorithms used in simulation environment route planning. Specifically, the paths, reward curves, and step curves generated by three algorithms, namely, DDPG, SAC, and HER-SAC, were observed and analyzed individually. The efficacy of the three algorithms could be determined by examining the reward curves.

Upon carrying out a comparative analysis of the reward curves pertaining to the DDPG and SAC algorithms, it becomes evident that both algorithms exhibit similar convergence rates over a given number of rounds. However, it is observed that the DDPG algorithm demonstrates a slower convergence, whilst the SAC approach showcases a more rapid convergence, ultimately leading to a superior final convergence outcome. To enhance the efficacy of incentive utilization, the HER algorithm was incorporated to optimize the SAC algorithm. Upon analyzing the reward curves of the SAC and HER-SAC algorithms, it becomes evident that the optimized algorithm shows a significant reduction in the number of rounds from the start of convergence to full convergence compared with the pre-optimized algorithm, hence enhancing the pace of convergence. Simultaneously, the ultimate convergence effect is enhanced compared with the pre-optimization state.

Based on empirical validation, it can be determined that the optimized algorithm produces superior results in the field of path planning. The effective navigation of mobile robots in real-world settings can be greatly enhanced by addressing the path-planning challenge in complex and unfamiliar environments. From the results obtained from the aforementioned trials, it can be inferred that the method under investigation demonstrates effective path-planning capabilities inside 2D environments. Hence, the proposed methodology can be extended to encompass other 2D environments featuring diverse sorts of obstacles. The algorithm’s effectiveness in real-time applications is diminished due to its reliance on learning from past knowledge and subsequently planning global courses mostly through hindsight experience replay. In the meantime, the subsequent phase can be expanded to encompass the involvement of several robots in the process of multi-intelligent body route planning. This can be achieved via the implementation of collaborative decision making, effective communication and collaboration among the robots, the utilization of group intelligence, and the ability to adapt to dynamic environmental conditions. Hence, the experiment exhibits three primary constraints: firstly, the absence of real-time path planning in the presence of dynamic obstacles; secondly, the absence of intricate obstacle path planning in a three-dimensional environment; and thirdly, the absence of multi-robot path planning. Hence, our forthcoming research endeavors will focus on this particular issue.

## 6. Conclusions

This study presented a novel approach to address the path-planning challenge encountered by mobile robots operating in unfamiliar and intricate surroundings. The suggested solution leverages deep reinforcement learning techniques to develop an algorithm for efficient path planning. The suggested algorithm, soft actor-critic (SAC), aims to enhance the learning and exploratory aspects of the system. Additionally, the usage rate of samples is improved via the incorporation of the hindsight experience replay (HER) algorithm. The simulation results provide evidence that the proposed algorithm is capable of efficiently determining the shortest path between the initial and target points. Furthermore, it demonstrates an enhanced convergence speed and effectiveness compared with alternative algorithms, resulting in faster and more accurate path finding.

In forthcoming times, there will be a greater utilization of algorithms that exhibit enhanced efficiency in the domain of path planning. Furthermore, the task of path planning will be executed across unfamiliar dynamic situations, encompassing both two-dimensional and three-dimensional spaces, in order to enhance its compatibility with real-world surroundings.

## Figures and Tables

**Table 1:** Various algorithms for path planning.

Path-Planning Algorithm	Dominance	Drawbacks	Complexity Theory
Q-learning	Simple and easy to implement, and discrete states and action spaces work better	Requires state–action–reward transition tables, and continuous state and action space issues do not apply	The complexity of the algorithm increases as the state and action space increases
Dyna-Q	Combines model learning and reinforcement learning to improve learning efficiency and stability	Requires additional computational and storage overhead to maintain the environment model	Higher time complexity, proportional to the number of model learnings and planned learnings
SARSA	Easy to implement, and better results for discrete state and action space problems	Not applicable for continuous state and action space problems	Depends on the size of the state–action space
DQN	Adaptation to continuous state and action space problems, expressive, and can be trained offline	Instability in the training process, long training time, and large amount of sample data	Depends on the level of dimensionality, neural network size, number of iterations, and buffer size
A3C	Parallelization of training, fast convergence, and adaptability	Training is unstable and requires a large number of training samples	Depends on the level of dimensionality, the size of the neural network, the number of iterations, and the size of the parallelized training
PPO	Fast convergence, efficient use of samples, and good algorithmic stability	Sensitive parameter selection and demanding training samples	Depends on the level of dimensionality, the size of the neural network, the number of iterations, and the number of trajectories sampled
SAC	Applicable to path planning in high-dimensional space, adaptive adjustment of parameters, and good robustness	Longer training time, large sample data size, and may require more computational resources for complex tasks	Depends on neural network size and number of iterations
HER-SAC	Solves sparse rewards, improves convergence and stability, and can handle complex tasks	Requires additional computation and storage overhead	Depends on the level of dimensionality, neural network size, number of iterations, and buffer size
DDPG	Applicable continuous state and action space problems with good convergence and performance	Sensitive to initial conditions, long training time, and need to tune hyperparameters	Depends on the size of the neural network and the number of iterations of training
DDPG-HL	Ability to deal with problems with multiple levels, improving learning efficiency and performance	Requires additional computational and storage overhead to maintain the hierarchy	Depends on the size of the neural network and the number of iterations of training and the number of levels

**Table 2:** Setting of action space and state space size.

Action Space	State Space	Action Bound
2	4	$\left[-1,\;1\right]$

**Table 3:** Relevant parameters of the HER-SAC algorithm.

Description	Parameter	Value
Actor network learning rate	actor_lr	3 × 10^−4^
Critic network learning rate	critic_lr	3 × 10^−3^
α parameter learning rate	alpha_lr	3 × 10^−4^
Hidden layer dimensions	hidden_dim	128
Discount factor	gamma	0.98
Soft update parameters	tau	0.005
Buffer size	buffer_size	10,000
Minimal size	minimal_size	500
Batch size	batch_size	64
Total training episodes	num_episodes	1000
Minimal training episodes	minimal_episodes	200
Target entropy	target_entropy	−0.1
Number of training samples	n_train	20

**Table 4:** Results of different path-planning algorithms.

Algorithm	Environment	Training Round	Start to Converge	Eventual Convergence	Path Length
DDPG	Map1	1000	200	/	21.26
SAC	Map1	1000	241	800	21.31
HER-SAC	Map1	1000	200	264	21.31
DDPG	Map2	1000	200	850	21.89
SAC	Map2	1000	204	632	21.90
HER-SAC	Map2	1000	213	300	21.90

## Data Availability

The datasets used and analyzed during the current study are available from the corresponding author upon reasonable request.
